# Integrating single-cell RNA-seq and bulk RNA-seq to explore prognostic value and immune landscapes of methionine metabolism-related signature in breast cancer

**DOI:** 10.3389/fgene.2024.1521269

**Published:** 2025-01-14

**Authors:** Yanxian Gao, Ziyu Feng, Hailong Zhao, Xinghai Liu, Muyu Zhu, Xiafei Yu, Xiaoan Liu, Xian Wu, Jing Tao

**Affiliations:** ^1^ Breast Disease Center, The First Affiliated Hospital of Nanjing Medical University, Nanjing, Jiangsu, China; ^2^ Department of General Surgery, Huangyuan People’s Hospital, Xining, Qinghai, China; ^3^ Department of General Surgery, The Fourth Affiliated Hospital of Nanjing Medical University, Nanjing Medical University, Nanjing, Jiangsu, China

**Keywords:** APOC1 gene, methionine metabolism, breast cancer, single-cell sequencing (scRNA-seq), prognostic signature, tumor immune microenvironment (TIME)

## Abstract

**Background:**

Neoadjuvant, endocrine, and targeted therapies have significantly improved the prognosis of breast cancer (BC). However, due to the high heterogeneity of cancer, some patients cannot benefit from existing treatments. Increasing evidence suggests that amino acids and their metabolites can alter the tumor malignant behavior through reshaping tumor microenvironment and regulation of immune cell function. Breast cancer cell lines have been identified as methionine-dependent, and methionine restriction has been proposed as a potential cancer treatment strategy.

**Methods:**

We integrated transcriptomic and single-cell RNA sequencing (ScRNA-seq) analyses based on The Cancer Genome Atlas (TCGA) database and Gene Expression Omnibus (GEO) datasets. Then we applied weighted gene co-expression network analysis (WGCNA) and Cox regression to evaluate methionine metabolism-related genes (MRGs) in BC, constructing and validating a prognostic model for BC patients. Immune landscapes and immunotherapy were further explored. Finally, *in vitro* experiments were conducted to assess the expression and function of key genes APOC1.

**Results:**

In this study, we established and validated a prognostic signature based on eight methionine-related genes to predict overall survival (OS) in BC patients. Patients were further stratified into high-risk and low-risk groups according to prognostic risk score. Further analysis revealed significant differences between two groups in terms of pathway alterations, immune microenvironment characteristics, and immune checkpoint expression. Our study shed light on the relationship between methionine metabolism and immune infiltration in BC. APOC1, a key gene in the prognostic signature, was found to be upregulated in BC and closely associated with immune cell infiltration. Notably, APOC1 was primarily expressed in macrophages. Subsequent *in vitro* experiments demonstrated that silencing APOC1 reduced the generation of tumor-associated macrophages (TAMs) with an M2 phenotype while significantly decreasing the proliferation, invasion, and migration of MDA-MB-231 and MDA-MB-468 breast cancer cell lines.

**Conclusion:**

We established a prognostic risk score consisting of genes associated with methionine metabolism, which helps predict prognosis and response to treatment in BC. The function of APOC1 in regulating macrophage polarization was explored.

## 1 Introduction

Breast cancer is the most prevalent cancer among women and the second leading cause of cancer-related mortality (Siegel et al., 2024), characterized by significant tumor heterogeneity, which poses numerous challenges for clinical treatment. Currently, the treatment strategy for breast cancer has shifted towards a multimodal approach (Coles et al., 2024), incorporating surgical intervention, chemotherapy, radiotherapy, endocrine therapy, anti-HER2 targeted therapy, and immunotherapy. Despite the widespread use of these methods in clinical practice, some patients still do not derive benefits from them. Prognosis is primarily assessed through factors such as tumor size, molecular subtype, lymph node status, mutations in high-risk genes, and the presence of distant metastases. However, these traditional assessment methods often fail to adequately predict patient survival or recurrence risks, resulting in delays in timely and effective treatment for high risk patients (Leon-Ferre and Goetz, 2023). Consequently, there is an urgent need to develop more precise prognostic tools and biomarkers to better identify high risk patients (Rizzo and Ricci, 2022), facilitating personalized predictions and precision therapies for breast cancer, ultimately aiming to improve treatment outcomes and patient survival rates.

Recent advances in immunotherapy have brought new hope for breast cancer treatment (Yan et al., 2022). The IMpassion130 trial demonstrated that the combination of the PD-L1 inhibitor atezolizumab with nab-paclitaxel significantly prolonged the overall survival (OS) of PD-L1-positive patients with advanced triple-negative breast cancer (TNBC) (Ahmed et al., 2020). Additionally, the KEYNOTE-355 trial confirmed that pembrolizumab in combination with chemotherapy significantly extended the OS in patients with a PD-L1 CPS ≥10, leading to FDA approval of pembrolizumab as a first-line treatment for PD-L1-positive advanced TNBC (Cescon et al., 2024). However, despite PD-L1 being the most widely used biomarker for immunotherapy efficacy, numerous studies have revealed that even PD-L1-negative patients may benefit from immunotherapy. This indicates the current need for more precise biomarkers to accurately identify patients who can truly benefit from immune checkpoint blockade therapy.

In parallel, metabolic reprogramming has increasingly been recognized as a hallmark of malignant tumors, enabling cancer cells to utilize diverse nutrients, generate energy, and synthesize essential biomacromolecules (Faubert et al., 2020). The nutrient metabolism of cancer cells, particularly amino acid metabolism, differs significantly from that of normal cells. Amino acids play crucial roles in cancer cells, serving not only as nutrients and signaling molecules but also regulating gene transcription and epigenetic modifications. Cancer cells typically alter amino acid metabolism to support proliferation, resist apoptosis, promote metastasis, and adapt to hypoxic environments (Liu et al., 2024). Studies have shown that the metabolism of glutamine/glutamate, cystine/cysteine, asparagine/aspartate, methionine, glycine, serine, and tryptophan differs from that in normal cells, with abnormal regulation of amino acid interconversion and transport. Amino acid metabolism not only plays a critical role in shaping the tumor microenvironment (TME) and facilitating immune evasion. For instance, many tumor cells upregulate IDO expression, leading to the breakdown of tryptophan into kynurenine, which suppresses effector T-cell activity and promotes the expansion of regulatory T cells (Tregs), thereby driving immune evasion (Stone and Williams, 2023). Additionally, tumor cells increase serine and glycine metabolism, which affects macrophage polarization and weakens T-cell function, further reducing the efficacy of immunotherapy (Shan et al., 2022). Based on these characteristics, targeting specific amino acids to modulate their metabolic pathways has emerged as a potential adjunctive therapeutic strategy for cancer cells (Chen et al., 2024).

Methionine (Met) is converted to S-adenosylmethionine (SAM) by methionine adenosyltransferase (MAT), participating in several critical biochemical processes such as redox maintenance, polyamine synthesis, and providing methyl groups for DNA, RNA, and histone methylation (Martinez et al., 2017). Many types of tumor cells are methionine-dependent, meaning they cannot survive in the absence of methionine, even when its precursor homocysteine (Hcy) is available. This reflects the metabolic reprogramming of methionine metabolism in tumors. Dietary restriction of methionine, an essential amino acid, may affect cancer treatment outcomes by modulating the one-carbon metabolic pathway (Lu and Luo, 2023). Studies have shown that methionine restriction can disrupt tumor metabolic pathways and enhance therapeutic responses in RAS-driven cancer models, such as colorectal cancer and soft tissue sarcoma (Gao et al., 2019). The metabolic reprogramming of methionine may affect the differentiation and functionality of various immune cells within the TME (Wang and Zou, 2020). *In vitro* studies have shown that the demand for methionine in lipopolysaccharide (LPS)-induced M1 macrophages arises from exogenous uptake. Exogenous methionine serves as a primary methyl donor for the generation of SAM and subsequent methylation reactions, which are crucial for the production of IL-1β. Furthermore, methionine significantly enhances the secretion of TNF-α from macrophages while reducing Arg1 activity, indicating that methionine promotes the M1 polarization phenotype of macrophages (Zhang et al., 2023). It can be inferred that tumor cells deplete methionine, leading to decreased levels of this amino acid in the TME, thereby inhibiting M1 polarization in macrophages and diminishing the anti-tumor efficacy of tumor-associated macrophages (TAMs), ultimately facilitating tumor progression. Tumor cells compete with T cells for methionine via SLC43A2, resulting in metabolic and epigenetic impairment of T cell function and weakening tumor immunity (Peng et al., 2023). Mahesh Pandit et al. found that methionine consumption by cancer cells progressively upregulates PD-1 expression in CD4^+^ T cells, promoting tumor immune evasion (Pandit et al., 2023; Siska and Rathmell, 2015; Wang et al., 2019). Recent studies have also demonstrated that methionine deficiency enhances anti-tumor immunity by altering the m6A methylation of immune checkpoint transcripts (Li et al., 2023).

In summary, the reprogramming of methionine metabolism not only impacts tumor cell survival and proliferation but may also influence the effectiveness of cancer therapies by modulating immune cell function. In this study, we integrated single-cell RNA sequencing and bulk RNA sequencing to construct and validate a reliable methionine metabolism-related feature model for predicting survival in breast cancer patients. Subsequently, we explored the potential relationships between this feature, immune infiltration, and immune checkpoint expression. Additionally, we evaluated the model’s predictive potential for responses to immunotherapy and chemotherapy.

## 2 Materials and methods

### 2.1 Data acquired

We employed TCGAbiolinks to download and process BC transcriptomic data from the TCGA database, ultimately including 1,088 BC samples in our analysis. The data were provided in TPM format and were converted to log2 for subsequent analysis. Additionally, we downloaded the GSE58812 dataset (n = 107) from the GEO database as an external independent validation cohort. We also obtained scRNA-seq data for four untreated BC samples from the GEO database with the accession number GSE161529. We used the GeneCards database as a source for MRGs, ultimately selecting 1,639 MRGs with a relevance score exceeding the median score for further investigation (Supplementary Table S1).

### 2.2 ScRNA-seq data analysis

We performed quality control on the ScRNA-seq data using the “Seurat” R package and analyses were conducted using R software (version 4.2.3). Low-quality cells with less than 500 or over 10,000 expressed genes, or over 25% unique molecular identifiers (UMIs) derived from the mitochondrial gene, or over 5% UMIs derived from the hemoglobin genes were removed. And we keep genes which were expressed in at least 3 cells.

Finally, A total of 20,233 cells and 22,315 genes were retained for further exploration. We used harmony to correct for batch effects across datasets. We first applied the LogNormalize method to normalize the remaining data, correcting for differences in total expression across cells. Top 2,000 highly variable genes were identified using the FindVariableFeatures function. Following this, we applied the ScaleData function to scale the data, reducing discrepancies in gene expression levels. Finally, we performed principal component analysis (PCA) on these highly variable genes to reduce dimensionality. Cell clusters were identified using the FindClusters function (resolution = 0.4) (Petegrosso et al., 2020). Subsequently, the FindAllMarkers function was employed to identify marker genes for each cluster, with a log fold change threshold set at 0.25. Finally, the clusters were annotated to known cell types using CellMarker 2.0 based on the identified marker genes (Jiang et al., 2021; Hu et al., 2023).

### 2.3 AUCell

Univariate Cox regression analysis identified 195 prognosis-related MRGs (Supplementary Table S6). Next, using the AUCell R package on the scRNA-seq dataset, the AUC value for each cell was calculated based on the selected MRGs, with higher gene expression leading to higher AUC values. Cells were then divided into high and low AUC groups based on the median AUC score, and visualization was performed using the “ggplot2” R package.

### 2.4 ssGSEA analysis

single-sample Gene Set Enrichment Analysis (ssGSEA) was used to determine the MRG score for each TCGA-BC sample, and the samples were divided into two groups based on the median score.

### 2.5 WGCNA analysis

Weighted Gene Co-expression Network Analysis (WGCNA) was used to describe gene co-expression patterns across multiple samples. We applied the “WGCNA” R package to identify gene modules associated with MRG scores in breast cancer (BC). The module most significantly correlated with the glutamine metabolism was selected for further analysis.

### 2.6 Construction and validation of MRGs-based prognostic signature

We used the breast cancer dataset from TCGA as the training set to construct the prognosis model and utilized the GSE58812 dataset as an external validation set for verification. We combined LASSO (Least Absolute Shrinkage and Selection Operator) with the Cox proportional hazards regression model to identify the model genes and their corresponding risk coefficients. Subsequently, by utilizing the “glmnet” R package, we constructed a risk feature related to MRGs that can predict the survival of BC patients in the training set. We calculated risk scores for each breast cancer patient using the algorithm and categorized patients into two subgroups based on the median risk score. Additionally, we assessed the prognostic value of the risk model in the testing set and an external validation set (GSE20711). Kaplan-Meier survival curves were plotted, and log-rank tests were conducted to evaluate the statistical significance of the prognosis. The accuracy of the risk model in predicting overall survival for BRCA patients was assessed using receiver operating characteristic (ROC) curves. To predict 1-, 3-, and 5-year survival of BC patients, we further developed a nomogram by combining risk scores with independent prognostic factors such as age and stage. ROC curves and calibration curves were used to evaluate the predictive accuracy of the nomogram.

### 2.7 Characterization of the immune microenvironment

To evaluate immune cell infiltration characteristics between two groups, we employed the “CIBERSORT” and “Xcell” R packages to quantitatively analyze the infiltration levels of various immune cells using the LM22 signatures. The relative abundances of stromal, immune, and tumor cells were assessed via the “ESTIMATE” algorithm, and these values were compared across different risk categories (Yoshihara et al., 2013). The expression of immune checkpoints reflects the immunosuppressive status within the tumor microenvironment, providing insights into how tumors evade immune surveillance. We compared the expression levels of established immune checkpoint genes between the two groups.

To predict the likelihood of response to immunotherapy, we utilized the TIDE algorithm to determine tumor immune dysfunction and exclusion scores, and downloaded Immunophenoscores (IPS) from The Cancer Immunome Atlas (TCIA) database (https://tcia.at/home). Additionally, we conducted a correlation analysis between APOC1 and immune cell infiltration using the Tumor Immune Estimation Resource (TIMER) website (http://timer.cistrome.org/).

### 2.8 Functional enrichment analysis

We used “clusterprofile” r packages to perform Gene Ontology (GO) enrichment analysis andGene Set Enrichment Analysis (GSEA). GSEA on APOC1 was performed using LinkedOmics website (https://www.linkedomics.org).

### 2.9 Cell culture

Human monocytic cell lines (THP-1) and Human BC cell lines (MDA-MB-231 and MDA-MB-468) were purchased from Cell Bank of Type Culture Collection of Chinese Academy of Sciences (Shanghai Institute of Cell Biology of the Chinese Academy of Sciences). THP-1 cells were cultured in RPMI 1640 medium supplemented with 10% fetal bovine serum (FBS) and incubated in sterile culture flasks at 37°C in a humidified incubator with 5% CO₂. The MDA-MB-231 and MDA-MB-468 cell lines were maintained in high-glucose DMEM supplemented with 10% FBS under similar conditions.

### 2.10 Macrophage generation

THP-1 cells line were pretreated with 100 ng/mL PMA for 48 h to generate M0 macrophages. M0 macrophages were co-cultured with MDA-MB-231 and MDA-MB-468 for 48 h in a 6-well transwell co-cultivation system, allowing them to be influenced by the secreted factors from the tumor microenvironment, thereby acquiring a TAM-like phenotype.

### 2.11 Transfection

The siRNAs targeting APOC1 were purchased from Tsingke Biotechnology (Beijing, China) and transfected using Lipofectamine 3000 according to the manufacturer’s instructions. After transfection, the cells were cultured for an additional 24 h, and gene knockdown efficiency was evaluated using RT-qPCR.

### 2.12 EdU

To evaluate the proliferation ability of MDA-MB-231 and MDA-MB-468 tumor cells after co-culture with macrophages, the 5-Ethynyl-2′-deoxyuridine (EdU) incorporation assay was performed using an EdU assay kit (BeyoClick™ EdU Cell Proliferation Kit with Alexa Fluor 488, Beyotime, China) according to the manufacturer’s protocol. The treated tumor cells were seeded at a density of 5 × 10^4^ cells per well in 96-well plates. After the co-culturing, EdU was added to the tumor cells, followed by incubation according to the protocol. After incubation, the cells were fixed with 4% paraformaldehyde (PFA) for 15 min, and then permeabilized with 0.5% Triton X-100 for 10 min. Next, the click reaction solution was added to the wells and incubated at 37°C for 30 min in the dark. Subsequently, the cells were stained with Hoechst 33342, and excess dye was washed away with PBS. Finally, the proliferation of tumor cells after co-culturing was assessed by analyzing the fluorescence signals using THUNDER Imaging Systems.

### 2.13 Transwell assay

To assess the invasion abilities of the cells, we conducted a Transwell experiment. Matrigel, diluted in serum-free medium, was added to the upper chamber to simulate the extracellular matrix. In the upper chamber, 1 × 10^4^ cells were seeded per well in serum-free medium, while the lower chamber contained 600 μL of complete medium. After incubating for 24 h at 37°C, the upper chamber was gently swabbed with a cotton swab to remove non-invasive cells. The cells that invaded through the membrane were then fixed with 4% paraformaldehyde for 15 min, stained with 0.1% crystal violet, and counted under a light microscope after washing off excess stain.

### 2.14 Wound healing assay

MDA-MB-231 and MDA-MB-468 cells co-cultured with macrophages were seeded in 6-well plates at a density of 1 × 10^6^ cells per well and cultured until they reached 90% confluence. A straight line was scraped down the center of the wells using a 200 μL pipette tip, and any unattached cells and debris were gently washed away twice with PBS. Images of the scratch wounds were captured at 0 h and 48 h, and the width of the scratches was measured using ImageJ software.

### 2.15 RT-qPCR

Total RNA was extracted from cell lines using SteadvPure RNA Extraction Kit (Accurate Biology, China) according to the manufacturer’s instructions. Subsequently, cDNA was synthesized using the PrimeScript™ RT Kit (R232-01, Vazyme). Real-time quantitative PCR (RT-qPCR) was performed using SYBR Green Master Mix (Q111-02, Vazyme), and expression levels were quantified using the 2^−ΔΔCT^ method. All samples were tested in triplicate. The primer sequences used in the RT-qPCR are listed in Supplementary Table S2 and were provided by Tsingke Biotech (Beijing, China).

### 2.16 Flow cytometry

For the macrophage suspension, antibodies (CD163 from Biolegend, United States) were added and incubated at 4°C for 30 min. After washing the labeled cells twice, they were resuspended in 200 μL of flow cytometry buffer and analyzed using a flow cytometer (FACS Calibur, BD Biosciences, United States), with data processing performed using CytExpertsoftware and FlowJo_v10.8.1.

### 2.17 Statistical analyses

All statistical analyses were performed using R software (version 4.2.3) or GraphPad Prism (version 8.0.2). Correlation analyses were conducted using Spearman or Pearson methods as appropriate. Data from three independent experiments are presented as mean ± standard deviation (SD). Differences between groups were assessed using the Wilcoxon test or unpaired *t*-test. P-value of <0.05 was considered statistically significant (*p < 0.05; **p < 0.01; ***p < 0.001).

## 3 Results

### 3.1 Myeloid cells were identified to exhibit higher MRG score via scRNA-seq analysis of breast cance

**FIGURE 1:**
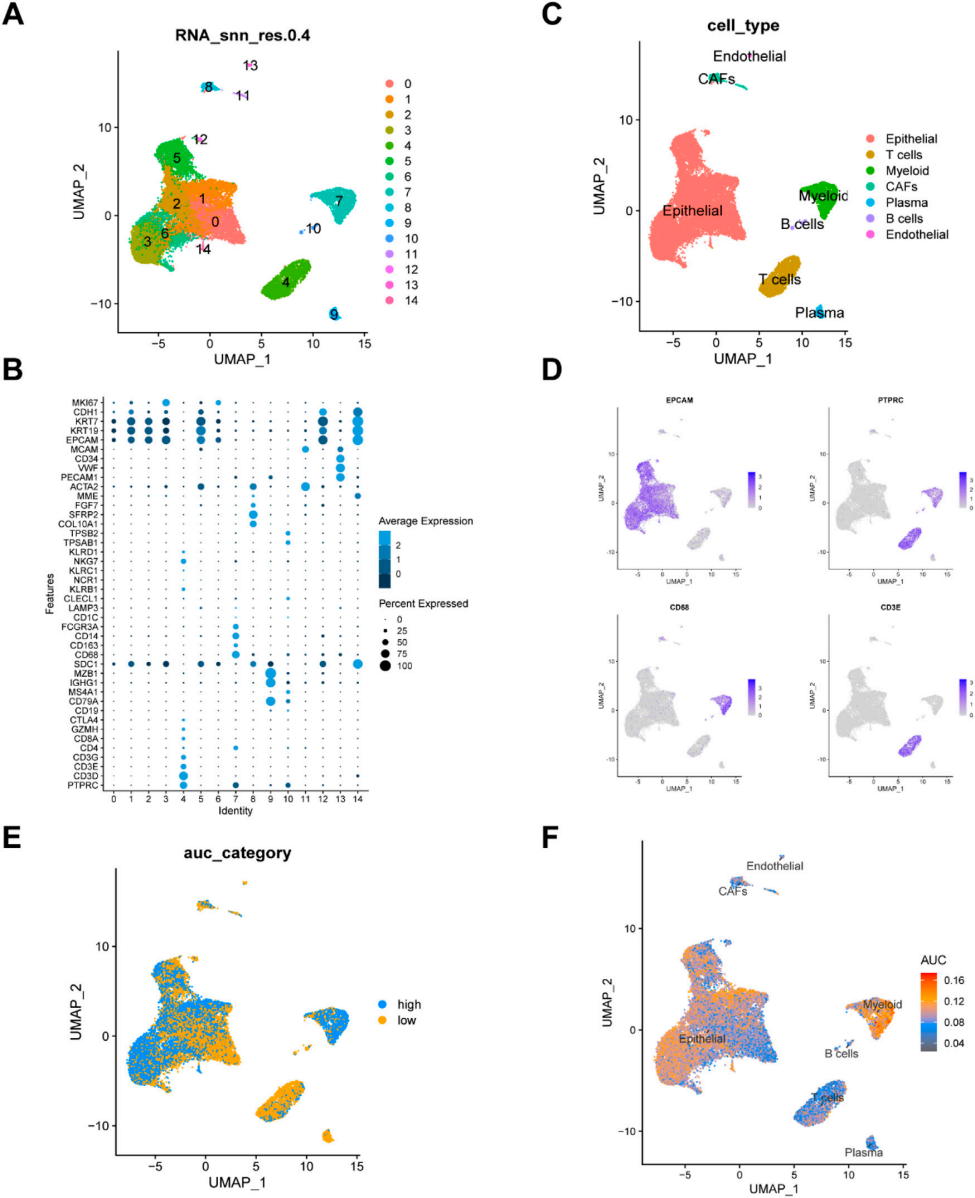
Analysis of single-cell RNA sequencing data and identification of differentially expressed genes. **(A)** UMAP visualization of dimension reduction cluster analysis results. A total of 25,932 cells were divided into 15 clusters. **(B)** The expression of cell type marker genes. **(C)** UMAP visualization of major cell types. **(D)** Depicting the expression of EPCAM, PTPRC,CD68, CD3E genes. **(E, F)** All cells were scored according to methionine metabolism-related genes (MRGs) and were divided into high and low groups.

A total of four breast cancer single-cell sequencing samples were included in this study (Supplementary Figure S1A). We performed quality control on the single-cell dataset by setting limits on the number of features (nFeature), total counts (nCount), and the percentages of mitochondrial genes, ribosomal genes, and red blood cell genes (Supplementary Figure S1B). As seen in Supplementary Figure S1C, sequencing depth and total intracellular sequences exhibit a strong positive correlation (R = 0.88). We found several clusters were composed one sample, suggesting there were potential batch effects, thus we corrected the batch effects using Harmony (Supplementary Figures S1D, E). A total of 25,932 cells were divided into 14 clusters (Figure 1A). We then identified 7 cell types based on marker genes for future analysis (Figure 1B), including endothelial cells (cluster 13), epithelial cells (clusters 0, 1,2,3,5,6,12,14), B cells (clusters 10), T cells (clusters 4), myeloid cells (cluster 7), fibroblasts (cluster 8,11), and plasma (cluster 9) (Figures 1C, D). To explore the expression characteristics of MRGs, we analyzed the MRGs activity of each cell using the “AUCell” R package. All cells were assigned an AUC score corresponding to MRGs, with cells expressing more MRGs showing higher AUC values. The results indicated that myeloid cells exhibited higher AUC values (Figure 1E). Cells were divided into high methionine-AUC and low methionine-AUC groups by the median AUC score (Figure 1F).

### 3.2 WGCNA identified gene modules associated with methionine metabolism and LASSO cox regression analysis developed a prognostic model

**FIGURE 2:**
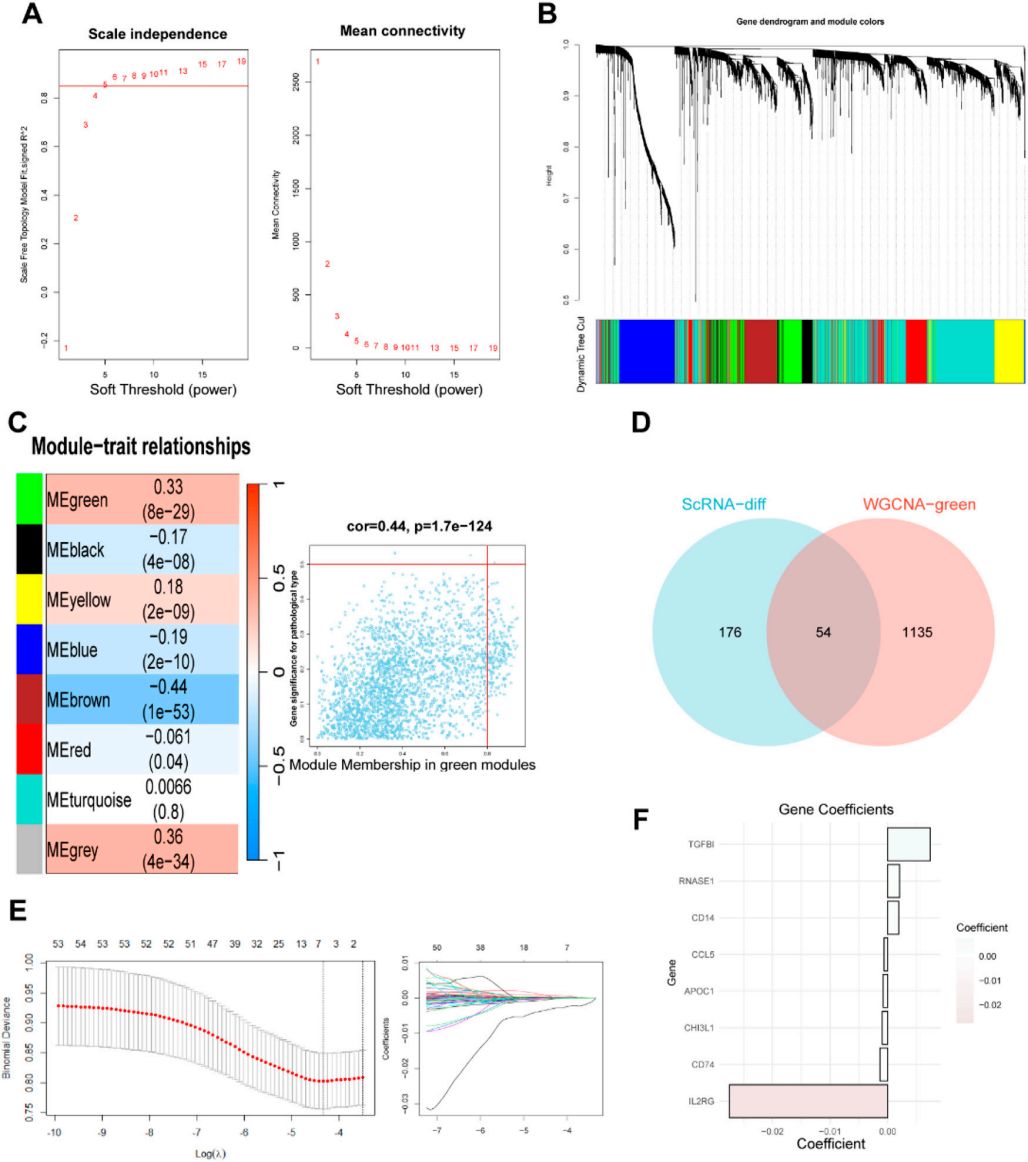
Weighted Co-Expression Network Analysis and identification of MRGs to establish a risk signature. **(A–C)** Weighted Co-Expression Network Analysis. The green modules were most associated with methionine metabolism, of which 2,783 genes were extracted. **(D)** The intersection of genes obtained in ScRNA-seq and bulk-RNA analysis. **(E)** LASSO Cox regression analysis to develop the prognostic model. **(F)** Identification of eight model genes.

We used the WGCNA R package to explore gene sets that covary with methionine metabolism. As shown in Figure 2A, when the soft-thresholding power was set to 5, the data better conformed to a scale-free network, and the mean connectivity stabilized, making the data suitable for further analysis. A total of eight modules were obtained after merging modules with a similarity lower than 0.25 and setting the minimum number of modules to 100 (Figure 2B). We found that the green module, which contains 1,189 genes (Supplementary Table S3), was most strongly associated with methionine metabolism (COR = 0.33, *P* < 0.001) in non-gray modules (Figure 2C). To further investigate how MRGs relate to the prognosis of BC patients, we intersected the most relevant genes affecting glutamine metabolism activity obtained from both single-cell and bulk RNA-seq analyses, ultimately selecting 54 genes for subsequent analysis (Figure 2D). We then employing LASSO Cox regression analysis to develop a prognostic model in TCGA-BRCA (Figure 2E). Under the optimal regularization parameter, we ultimately selected eight model genes (CD74, RNASE1, CD14, CHI3L1, CCL5, TGFBI, APOC1, IL2RG). Of the eight genes used to construct the model, three were risk factors and five were protective factors (Figure 2F).

**FIGURE 3:**
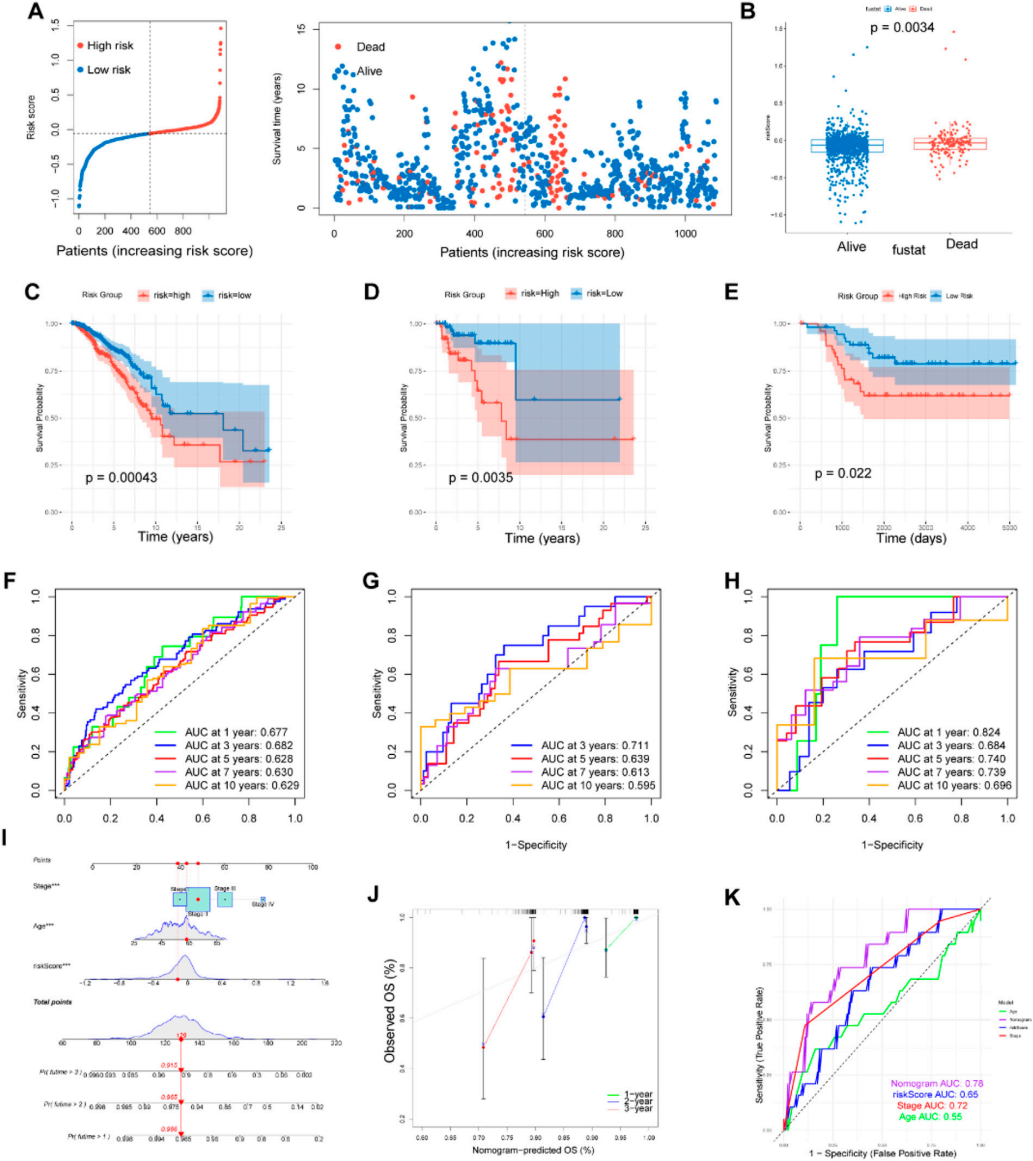
Construction and validation of prognostic model. **(A, B)** Distributions of risk scores and survival status in patients from TCGA. The risk scores of patients who died were higher than those of patients who lived. **(C–E)** Survival analysis in the patients form TCGA train cohort, GEO test cohort and TNBC cohort classified based on the median risk score (*P* < 0.05). **(F–H)** The area under the curve (AUC) values at 1, 3, 5, 7and 10-years for the TCGA train cohort, GEO test cohort and TNBC cohort. **(I)** Prognostic nomogram considering the risk score, age and stage to assess the risk of BC patients. **(J)** Calibration curves for the nomogram. **(K)** Prognostic ROC curves of age, stage, risk score and the nomogram in predicting 5-year OS.

The risk score for each sample was computed using the above formula where Coefi and Expi represent the coefficient and expression level of each model gene, respectively (Supplementary Table S4). Patients were divided into high risk and low risk groups with the median score as the threshold. The proportion of deceased patients in the high risk group was higher than that in the low risk group (Figure 3A). Additionally, patients who died during the follow-up period exhibited increased risk scores (Figure 3B). Kaplan-Meier analysis showed that patients with low risk had a much better overall survival (OS) rate (*p* = 0.00043) in the TCGA cohort (Figure 3C). Time-dependent ROC analysis revealed that the area under the curve (AUC) values for predicting 1-, 3-, 5-, 7- and 10-year OS were 0.677, 0.682, 0.628, 0.630 and 0.629, respectively (Figure 3F).

### 3.3 Validation of the MRGs-based prognostic signature for BC patients

**FIGURE 4:**
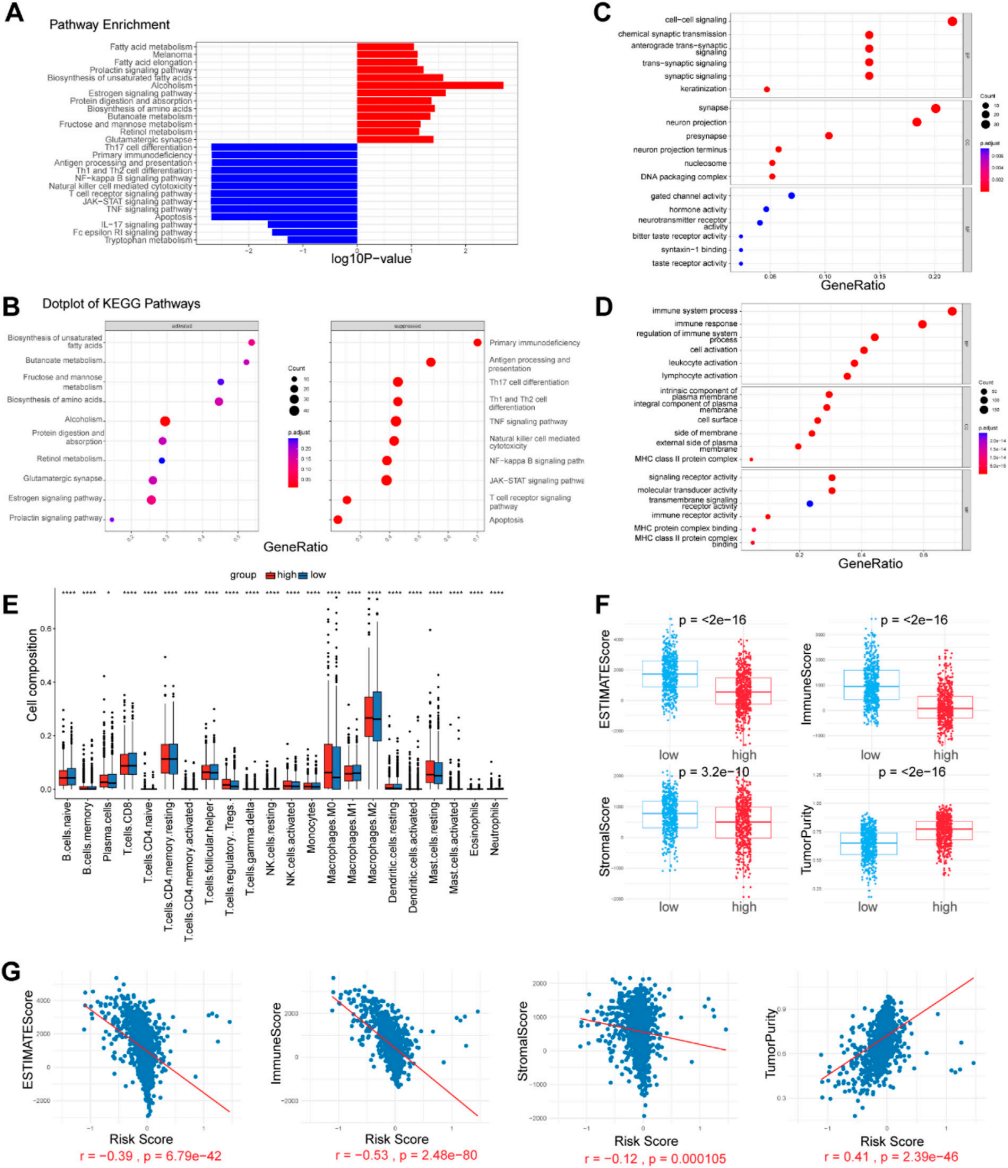
Enrichment analysis and immune microenvironment patterns. **(A, B)** KEGG pathways enriched in the high- and low-risk patients, as determined by GSEA. **(C, D)** GO analysis on both upregulated and downregulated genes, revealing differentially activated pathways. **(E)** Comparison of 22 tumor-infiltrating immune cells (TIICs) levels calculated by CIBERSORT analysis between the two risk groups. **(F, G)** Correlation analysis of immune cell infiltration levels and the risk score. The immune score, ESTIMATE score, Stromal score and tumor purity were estimated by the ESTIMATE algorithm.

To validate the reliability of the MRGs-based prognostic signature developed from the training set, we used a GEO cohort (GSE58812) as an external validation set. Survival analysis and ROC curve analysis were performed on the GEO cohort. Using the same risk score calculation formula and median risk score, patients in the validation cohort were divided into low risk and high risk groups. In the survival analysis, patients in the low risk group demonstrated higher OS rate compared to those in the high risk group (*p* = 0.0035), consistent with the results from the training set (Figure 3D). The AUC values for the external validation GEO cohort were 0.711, 0.639, and 0.613 at 3, 5, and 7 years, respectively, further confirming the prognostic value of our signature (Figure 4G). Limiting methionine has been shown to effectively inhibit the migration and invasion of TNBC cells in both *in vivo* and *in vitro* experiments. Survival analysis and ROC curve analysis conducted on the TNBC samples (n = 121) from TCGA cohort produced favorable results, reinforcing the prognostic significance of our findings (Figures 3E, H).

To enhance patient risk assessment and inform future treatment strategies, we developed a prognostic nomogram that integrates the risk score along with two additional clinicopathologic factors—age and stage. This approach allows for a more precise quantification of risk in breast cancer patients (Figure 3I). The calibration plot demonstrated the stable performance of the nomogram (Figure 3J). Moreover, our nomogram had better predictive accuracy than the AJCC staging system (AUC at 5 years: 0.78 versus 0.72) (Figure 3K).

### 3.4 Biological pathway analysis reveals key mechanisms for poor prognosis in high risk breast cancer patients

To explore the underlying mechanism that could lead BC patients in the high risk group to a poor prognosis, we performed differential expression analysis between the two risk groups and identified 465 differentially expressed genes (DEGs) (adjusted *p*-value <0.05, |Log2-fold change| > 1) (Supplementary Table S5). Gene Set Enrichment Analysis (GSEA) revealed that the Estrogen signaling pathway, Biosynthesis of unsaturated fatty acids, Retinol metabolism, Fatty acid elongation, Melanoma, Fatty acid metabolism, and Glutamatergic synapse were significantly enriched in high risk group. The low risk group exhibited enrichment in pathways related to Antigen processing and presentation, Th1 and Th2 cell differentiation, T cell receptor signaling, NF-kappa B signaling, JAK-STAT signaling, Natural killer cell-mediated cytotoxicity, and TNF signaling (Figures 4A, B). Additionally, we conducted GO analysis on both upregulated and downregulated genes. The results indicated that upregulated genes were significantly enriched in pathways related to signal transduction and DNA replication-dependent nucleosome assembly. In contrast, downregulated genes showed significant enrichment in immune system processes, regulation of immune system processes, and immune receptor activity (Figures 4C, D). These findings suggest that the established signature is closely related to immune activities, fatty acid metabolism, and signal transduction, thereby enabling it to predict the survival of BC patients.

### 3.5 Differences in immune landscape between low-risk and high-risk breast cancer patients

Tumor-infiltrating immune cells (TIICs) are a crucial component of the tumor microenvironment (TME), and their absence can lead to poor responses to immune checkpoint blockade. We evaluated the infiltration levels of various TIICs between the two risk groups to uncover differences in the immune microenvironment. Immune cell infiltration levels were assessed in each sample using the CIBERSORT and xCell methods. We found that immune cell infiltration was overall higher in the low risk group than in the high risk group. Notably, NKT cells, M1 macrophages, CD4^+^ T cells, and CD8^+^ T cells were more abundantly infiltrated in the low risk group (*p* < 0.05). These cell types are widely recognized for their antitumor activities within the tumor microenvironment, suggesting a favorable prognosis. Conversely, Tregs, monocytes, M0 macrophages, M2 macrophages, and resting mast cells were more prevalent in the tumors of the high risk group (*p* < 0.05). However, immune cells with antitumor functions, including plasma cells, follicular helper T cells, and activated NK cells, were also more abundant in the high risk group (*p* < 0.05) (Figure 4E). We further analyzed the correlation between the abundance of TIICs and the risk score. According to the ESTIMATE algorithm, the low risk group exhibited higher stromal scores, immunological scores, and ESTIMATE scores (*P* < 0.05) (Figures 4F, G). The risk score was strongly negatively correlated with the immune score and positively correlated with tumor purity, indicating a higher overall immune level and immunogenicity within the TME of the low risk group.

### 3.6 Immune checkpoint analysis and immunotherapy response prediction

**FIGURE 5:**
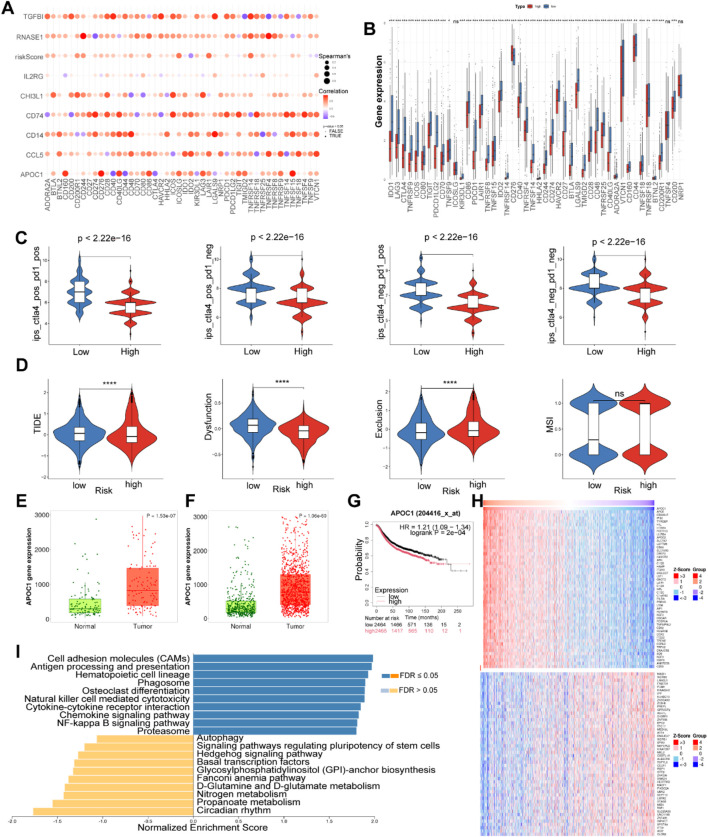
Correlation analysis of immune checkpoint in the TCGA cohort and expression analysis of APOC1. **(A)** Correlation analysis of eight model genes and immune checkpoint genes. **(B)** The expression levels of immune checkpoint genes between high and low risk groups. **(C)** Differences in ips score between high and low risk groups. **(D)** The difference in TIDE scores between high and low-risk groups. (*P < 0.05, **P < 0.01, ***P < 0.001, ****P < 0.0001). The ns indicates No significance. **(E)** Expression of APOC1 in normal and paired tumor tissues of BC. **(F)** Expression of APOC1 in normal and tumor tissues of BC. **(G)** The surviva analysis of APOC1 in Kaplan-Meier plotter. **(H)** Heatplot showed genes associated with APOC1. **(I)** GSEA of APOC1 using LinkedOmics.

We subsequently investigated the correlation between MRGs based signature and immune checkpoints, assessing its potential role in predicting responses to immunotherapy. The bubble plot illustrated the relationships between the model genes and 46 immune checkpoint genes (Figure 5A). Notably, TGFBI, RNASE1, CD74, CD14, CCL5, and APOC1 exhibited significant correlations with various immune checkpoint genes. We compared the expression levels of several immune checkpoint genes between the two groups. Most analyzed immune checkpoint genes, including IDO1, IDO2, LAG3, CTLA4, TNFRSF9, ICOS, CD80, PDCD1LG2, TIGIT, CD70, TNFSF9, and PDCD1, were markedly upregulated in the low risk group (*p* < 0.05). This suggests that patients with this tumor subtype may benefit from targeted therapies directed against the upregulated immune checkpoints. Conversely, only three immune checkpoint genes (ICOSLG, TNFSF4, and NRP1) did not show significant upregulation in the low risk group (Figure 5B).

Additionally, the Immune Phenotype Score (IPS) can be instrumental in identifying patients who are likely to respond to immunotherapy. In our study, the low risk group exhibited higher IPS scores compared to the high risk group, indicating that low risk patients may be more susceptible to anti-CTLA-4 and anti-PD-1 therapies, potentially deriving greater therapeutic benefits (Figure 5C). This observation may be attributed to increased infiltration of Tregs and elevated expression levels of PD1 and CTLA4 in the low risk group (Figure 5B).

To further predict whether patients may benefit from immunotherapy, we employed TIDE analysis to comprehensively assess the differences in the tumor microenvironment between high risk and low risk groups. Compared to the low risk group, the high risk group exhibited lower TIDE and Dysfunction Scores, alongside higher MDSC and Exclusion Scores (Figure 5D). These findings suggest that T cells in the high risk group can function to some extent; however, the elevated levels of MDSCs and the stronger exclusion mechanisms within the tumor microenvironment still indicate a state of immune suppression. Furthermore, the low levels of T cell infiltration in the high risk group may significantly contribute to tumor progression and poor prognosis. Although the low risk group demonstrates less suppression from MDSCs and exclusion mechanisms, allowing for easier T cell infiltration, the higher TIDE and Dysfunction Scores imply that immune evasion in this group primarily relies on T cell dysfunction, preventing effective tumor cell destruction (Jiang et al., 2018). This apparent contradiction between the immune escape observed in the low risk group and their favorable prognosis may reflect the predominant role of other non-immune factors, such as genetic mutations and tumor proliferation rates, in tumor progression within this cohort. Thus, these results underscore the complexity of the tumor microenvironment and its impact on responses to immunotherapy, highlighting the importance of considering multiple biological factors when designing immunotherapeutic strategies.

### 3.7 APOC1 is highly expressed in breast cancer and is associated with a poorer prognosis

In both paired and unpaired samples, the expression level of APOC1 was significantly higher in BC tissues compared to normal tissues (Figures 5E, F). The expression of the other seven model genes in normal and tumor tissues were presented in Supplementary Figures S1F–L. Kaplan-Meier survival analysis demonstrated that BC patients with high APOC1 expression had shorter overall survival times (*p* < 0.05) (Figure 5G). Subsequently, we identified genes associated with APOC1 (Figure 5H) and performed GSEA using LinkedOmics. As shown in the bar plot (Figure 5I), APOC1 exhibited a strong positive correlation with several critical signaling pathways involved in immune regulation and cellular processes, including cell adhesion molecules (CAMs), antigen processing and presentation, natural killer cell-mediated cytotoxicity, cytokine-cytokine receptor interaction, chemokine signaling pathway, and the NF-kappa B signaling pathway, suggesting that APOC1 may play a role in tumor initiation, progression, and metastasis by modulating immune responses and cellular processes within the tumor microenvironment.

**FIGURE 6:**
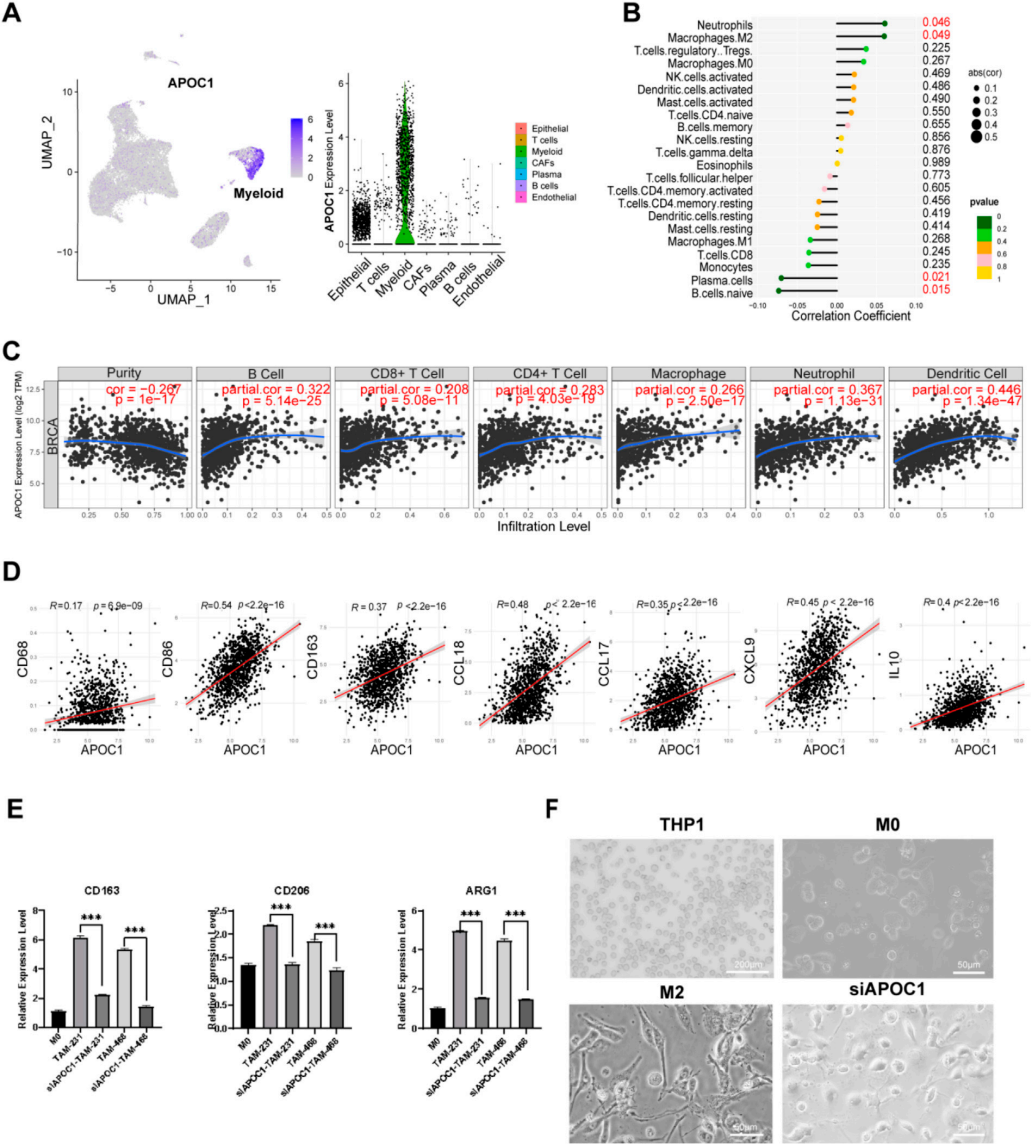
Inhibition of APOC1 of TAMs reduced M2 polarization of macrophages. **(A)** APOC1 was mainly expressed in macrophages through ScRNA-seq analysis. **(B)** APOC1 was closely related to infiltration of M2 macrophages among the immune infiltration cells. **(C)** The expression of APOC1 was significantly correlated with the infiltration level of various types of immune cells in BRCA. **(D)** APOC1 was significantly co-expressed with CD68, CD86, CD163, CCL18, CCL17, CXCL9, IL10 in BRCA. **(E)** The mRNA level of CD163, CD206, ARG1 were significantly downregulated in siAPOC1-TAMs via RT-qPCR experiment (*P < 0.05, **P < 0.01, ***P < 0.001). **(F)** The morphology of macrophage in THP1(×40, scale bar, 200 μm), M0, M2, siAPOC1-TAM(×200, scale bar, 50 μm).

ScRNA-seq analysis of BC samples revealed that APOC1 was almost exclusively expressed in macrophages (Figure 6A). Previous cross-tissue ScRNA-seq analyses have also confirmed that APOC1 is primarily expressed in macrophages. Correlation analysis between APOC1 expression and 22 types of immune cells showed that neutrophils and M2 macrophages were positively correlated with APOC1 expression, which is associated with poor prognosis, while plasma cells and naive B cells were negatively correlated (Figure 6B). Further assessment of APOC1 expression and immune infiltration levels in BC using TIMER revealed that APOC1 expression was positively associated with the infiltration levels of CD4^+^ T cells, CD8^+^ T cells, neutrophils, macrophages, and dendritic cells (Figure 6C). Additionally, APOC1 expression was significantly correlated with markers of macrophages (Figure 6D), including CD68 (r = 0.17, *P* = 6.93e-09), CD86 (r = 0.54, *P* = 1.77e-82), CD163 (r = 0.37, *P* = 2.21e-37), CCL18(r = 0.48, *P* = 4.20e-64), CCL17 (r = 0.35, *P* = 7.81e-32), CXCL9 (r = 0.45, *P* = 1.46e-54) and IL10 (r = 0.40, *P* = 5.71e-44). In summary, these findings indicate that APOC1 is predominantly expressed in macrophages, and its role in tumor immunity is likely mediated through its impact on macrophage function.

### 3.8 Inhibition of APOC1 of TAMs reduced M2 polarization of macrophages *in vitro* experiment

To further investigate the role of APOC1 in macrophage polarization, functional experiments were conducted using THP-1 cell lines. We silenced APOC1 expression using siRNA. Human THP-1 monocytes were differentiated into M0 macrophages through PMA treatment for 24 h, followed by co-culture with MDA-MB-231 and MDA-MB-468 cell lines for 48 h to induce the formation of tumor-associated macrophages (TAMs). Then we performed RT-qPCR to assess the expression of M2 phenotype-related genes in TAMs and si-APOC1 TAMs after co-culturing with MDA-MB-231 and MDA-MB-468 cell lines. Consistent with previous studies (Jiang et al., 2021; Ren et al., 2022; Rey-Giraud et al., 2012), we confirmed that macrophages co-cultured with cancer cells exhibited high expression of CD163, CD206, Arg1, and IL-10, markers indicative of an M2 phenotype. RT-qPCR analysis revealed that silencing APOC1 significantly reduced the expression of M2 macrophage markers CD163, CD206, ARG1, and IL-10 (Figure 6E). After co-culturing with tumor cells, macrophages typically adopt a spindle-like, elongated morphology, indicating M2 polarization. This change may also reflect their role in migration and extracellular matrix remodeling in the tumor microenvironment. Silencing APOC1 expression altered TAM morphology, causing them to lose the typical elongated M2-like shape (Figure 6F).

**FIGURE 7:**
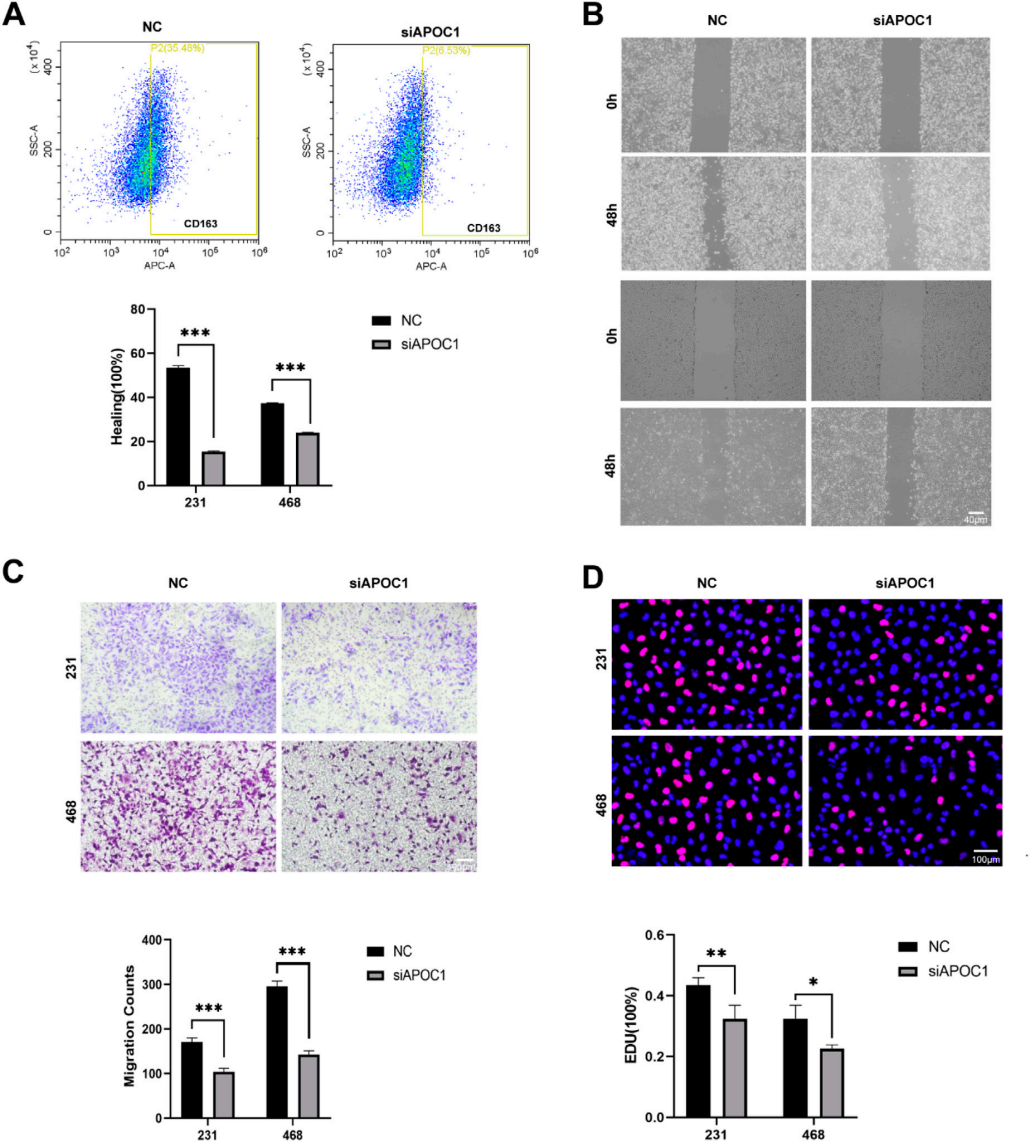
Inhibition of APOC1 of TAMs reduced BC progression *in vitro* experiment. **(A)** C163 were significantly downregulated in siAPOC1-TAMs via flow cytometry analysis. **(B)** Macrophages silencing APOC1 inhibited the migration of MDA-MB-231 and MDA-MB-468 cell lines via wound healing assay (×40, scale bar, 100 μm). **(C)** Macrophages silencing APOC1 inhibited the invasion ability of MDA-MB-231 and MDA-MB-468 cell lines via Transwell assays (×100, scale bar, 100 μm). **(D)** Macrophages silencing APOC1 inhibited the proliferation ability of MDA-MB-231 and MDA-MB-468 cell lines via EdU test (×100, scale bar, 50 μm). The experiments were performed in triplicate, and the data are presented as mean ± SD, *P < 0.05, **P < 0.01, ***P < 0.001 vs. control group.

Flow cytometry further confirmed that the expression of CD163 was markedly downregulated after silencing APOC1 (Figure 7A). These results indicate that *in vitro*-generated TAMs exhibit an M2 phenotype, and inhibiting APOC1 expression effectively prevents tumor cells from promoting the polarization of macrophages towards the M2 phenotype. This provides new insights into the mechanisms by which APOC1 regulates macrophage polarization within the tumor microenvironment and reveals its potential role in tumor-associated immune modulation.

### 3.9 Inhibition of APOC1 of TAMs reduced BC progression *in vitro* experiment

We further investigated the effects of APOC1-silenced macrophages on cancer cells by employing a co-culture system with the MDA-MB-231 and MDA-MB-468 cell lines. In the wound healing assay using the MDA-MB-231 and MDA-MB-468 cell lines, co-cultured macrophages significantly promoted the migration of tumor cells, while APOC1-silenced TAMs largely reversed this effect (Figure 7B). Similarly, Transwell assays demonstrated that APOC1-silenced TAMs suppressed the invasion capability of the tumor cells (Figure 7C). Furthermore, results from EdU proliferation assays indicated that, compared to the control group, APOC1-silenced TAMs effectively slowed down the proliferation of tumor cells (Figure 7D). These findings suggest that APOC1 plays a critical role in regulating TAM-mediated support for the proliferation, migration, and invasion of breast cancer cells. Thus, inhibiting APOC1 expression may help limit the progression of breast cancer.

## 4 Discussion

In this study, we constructed a robust survival risk signature based on methionine metabolism-related genes, which performed well in both internal (TCGA) and external (GEO) validation cohorts. Additionally, we developed a nomogram that integrates the prognostic model with clinical-pathological factors. This risk signature outperformed traditional features such as age and stage in predicting OS, demonstrating superior accuracy and discriminatory ability. Most of the included genes have been reported to play functional roles in various malignancies and are closely associated with immune cell infiltration.

Apolipoprotein C1 (APOC1), the smallest member of the apolipoprotein family, has been primarily studied in the context of lipid metabolism (Jong et al., 1999). Increasing evidence indicates that APOC1 is overexpressed in various cancers and is significantly associated with poor patient prognosis. However, the mechanisms of APOC1 in tumorigenesis, progression, and metastasis, particularly in tumor immune regulation, remain unclear. Further investigation into the function of APOC1 within the tumor immune microenvironment could provide valuable insights into the mechanisms underlying cancer progression and offer novel therapeutic opportunities. Previous studies have indicated that APOC1 serves as a prognostic marker for cervical cancer, ovarian cancer, and liver cancer (Shi et al., 2020; Yang et al., 2024), and it promotes glioblastoma tumorigenesis by inhibiting ferroptosis regulated by the KEAP1/NRF2 and CBS (Zheng et al., 2022). Recent research has also found that APOC1 facilitates the M2 macrophages polarization through ferroptosis, thereby remodeling the tumor immune microenvironment (Hao et al., 2022). However, there is currently limited information regarding the role of APOC1 in breast cancer. In this study, we investigated the role of APOC1 in BC by analyzing data from the TCGA and GEO databases, utilizing various platforms including LinkedOmics, TIMER, and Kaplan-Meier Plotter. Our results indicate that APOC1 expression is significantly upregulated in BC compared to normal tissues, and higher levels of APOC1 are associated with poorer patient prognosis. APOC1 is primarily expressed in tumor cells and macrophages and shows a positive correlation with the expression of genes such as CD68, CD86, CD163, CXCL17, CXCL8, and IL-10, suggesting that APOC1 may promote tumor progression by influencing macrophage polarization. Previous work by Jinhua Wang et al. has demonstrated *in vitro* that silencing APOC1 expression reverses the M2 polarization of tumor-associated macrophages (TAMs) co-cultured with renal cell carcinoma cells, while macrophages overexpressing APOC1 promote the metastasis of renal cell carcinoma through CCL5 (Ren et al., 2022). Building on this, our further *in vitro* experiments revealed that knocking down APOC1 in the M0 macrophages significantly reduced markers of M2 macrophages when co-cultured with 231 cell lines. These findings provide additional support for the hypothesis that APOC1 plays a role in breast cancer by promoting M2 polarization of macrophages. Many previous studies have shown that APOC1 may be involved in breast cancer progression and metastasis through mechanisms such as epithelial-mesenchymal transition (EMT) and the MAPK/JNK signaling pathway (Zhang et al., 2022). However, this study lacks a more in-depth exploration of the underlying mechanisms by which APOC1 exerts its effects.

## 5 Conclusion

In conclusion, our study developed a predictive model based on MRGs, demonstrating its effectiveness in forecasting OS and immunotherapy response in breast cancer patients. Additionally, through *in vitro* experiments, we explored the role of APOC1 in macrophage polarization. These findings provide valuable insights for the development of novel therapeutic strategies for breast cancer.

## Data Availability

The original contributions presented in the study are included in the article/Supplementary Material, further inquiries can be directed to the corresponding authors.
