# Supplementary figures and images for: Integrating single-cell RNA-seq and bulk RNA-seq to explore prognostic value and immune landscapes of methionine metabolism-related signature in breast cancer
Source: Front Genet. 2025 Jan 14;15:1521269. doi: 10.3389/fgene.2024.1521269 (PMC11772272; doi:10.3389/fgene.2024.1521269)

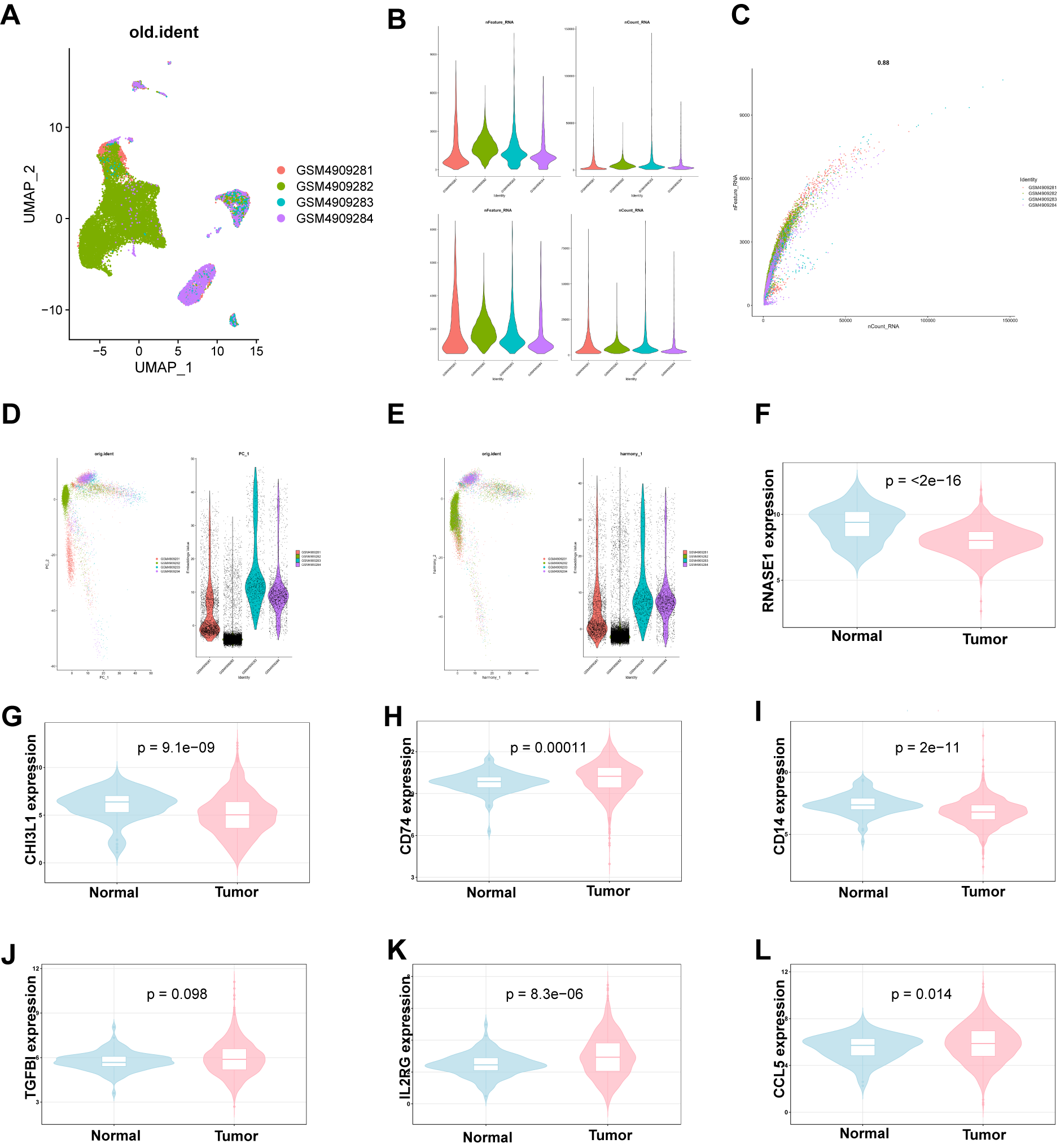

Supplement: Supplementary file 1 [file Image1.tif]
