# Supplementary material for: Integrating single-cell RNA-seq and bulk RNA-seq to explore prognostic value and immune landscapes of methionine metabolism-related signature in breast cancer
Source: Front Genet. 2025 Jan 14;15:1521269. doi: 10.3389/fgene.2024.1521269 (PMC11772272; doi:10.3389/fgene.2024.1521269)
Supplement: Supplementary file 2 [file DataSheet2.docx]

# Supplementary information

### Integrating single-cell RNA-seq and bulk RNA-seq to explore prognostic value and immune landscapes of methionine metabolism-related signature in breast cancer

Yanxian Gao^[[1]](#footnote-1)^^[[2]](#footnote-2)^†, Ziyu Feng^1†^, Hailong Zhao^[[3]](#footnote-3)^, Xinghai Liu^1^, Muyu Zhu^1^, Xiafei Yu1, Xiaoan Liu^1^*, Xian Wu1*, Jing Tao^[[4]](#footnote-4)^^[[5]](#footnote-5)^*

Supplementary Figure


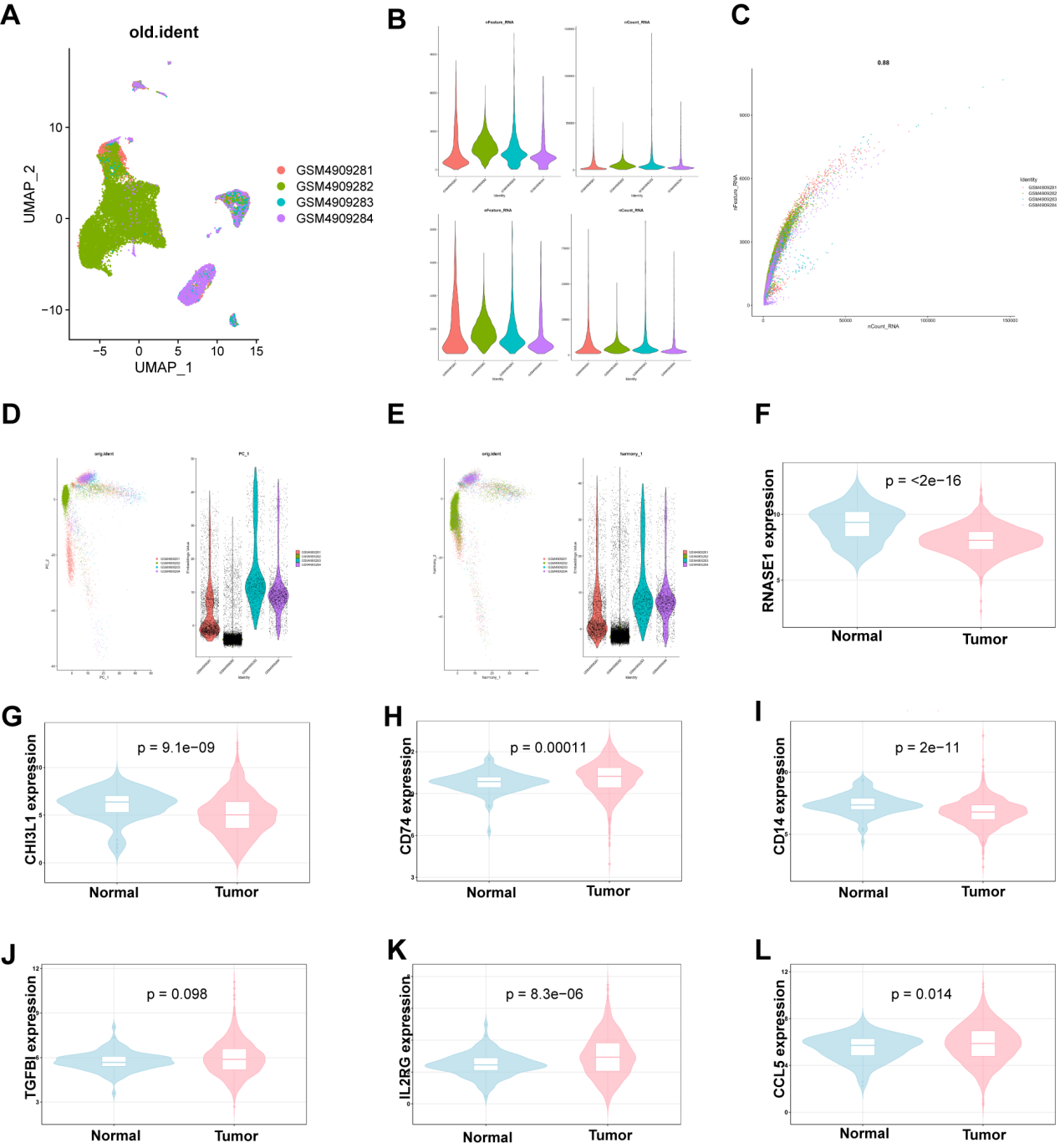


Supplementary Figure S1

(A)UMAP visualization of four breast cancer single-cell sequencing samples.

(B)Results of quality control analysis throughout nFeature and nCount.

(C)Illustration of the strong positive correlation (R = 0.88) between sequencing depth and total intracellular sequences.

(D-E)Display of the results after batch effect correction using Harmony.

(F-L)Expression of the other seven genes in normal and tumor tissues of BC from TCGA database.

1. Department of General Surgery, The Fourth Affiliated Hospital of Nanjing Medical University, Nanjing Medical University, Nanjing, Jiangsu, China [↑](#footnote-ref-1)
2. † These authors contributed equally. [↑](#footnote-ref-2)
3. Department of general surgery, Huangyuan people's Hospital, Xining, Qinghai province, China [↑](#footnote-ref-3)
4. Department of General Surgery, The Fourth Affiliated Hospital of Nanjing Medical University, Nanjing Medical University, Nanjing, Jiangsu, China [↑](#footnote-ref-4)
5. * To whom correspondence should be addressed: Jing Tao (Corresponding Author), Email: chnn123@163.com, Xian Wu(Corresponding Author), Email:wuxian126@ 126.com, Xiaoan Liu (Co-Corresponding Author), Email:liuxiaoan@126.com [↑](#footnote-ref-5)
